# Toxic Dermatoses

**Published:** 1904-04

**Authors:** Bernard Wolff

**Affiliations:** Atlanta, Ga.


### TOXIC DERMATOSES.*

BY BERNARD WOLFF, M.D.,

ATLANTA, GA.

A moment's consideration of the topic assigned me will discover its exceedingly wide scope—quite too extensive to be even partially encompassed in the time and space allowed me. It reaches far into the depth of pathologic conjecture and hypothesis in the direction to be taken by those who in the future shall seek for explanations of the mystery of disease. There can be no doubt but that toxins have an important influence upon the production of many disturbances of the integument, whether absorbed from without or from within, and acting upon the skin through the medium of the circulatory or nervous systems, one or both.

Their action is as yet presumptive and has not been definitely proven. The rationality of the belief is based upon the exposed position of the skin and upon its physiologic function as an excretory organ and the fact of its possessing an elaborate vascular, lymphatic, nervous and glandular arrangement, which renders its peculiarly susceptible to morbid influences.

It offers the medium of exhibiting outwardly many internal disturbances, for instance, the exanthemata, though we readily recognize that the skin is only secondarily involved, and what is visible upon it is but the eruption or breaking through of some agent which has caused a more or less violent disorder of the organs and structures which lie hidden beneath it.

Putting aside the exanthemata, including syphilis, there are certain skin eruptions occurring in the course of, or as a sequel to, rheumatism, gout, malaria, gonorrhoea, diphtheria, renal and hepatic diseases, influenza, septicemia, vaccination, antitoxin and tuberculin serum injections. These belong to the general class of neurotic erythematous and hemorrhagic eruptions.

Rheumatism is frequently accompanied by disturbances of the skin. The skin at certain localities, especially the face and eyes and lower extremities, becomes intensely itchy, red, and sometimes

*Read before Atlanta Society of Medicine, March 3, 1904.

swollen. The erythema is fugitive in character or may be absent except when produced by the fingers in scratching or rubbing. The itching is always present. These periodic outbursts of itching and redness are very noticeable in those of the arthritic habit, and may or may not accompany a frank attack of rheumatism. These outbreaks are prone to occur at change of season, though they may continue through summer or winter, subject to occasional aggravation. Occasionally the congestive erythema is so marked as to resemble scarlet fever, and with its acute onset may give rise to some perplexity as to its identity. Its subsequent course and lack of marked febrile reaction will generally serve to distinguish it from true scarlatina.

Purpuric eruption, the so-called peliosis or purpura rheumatica, belongs to the exudative erythemata and marks a high grade of cutaneous reaction to toxic influences.

How far the retention of toxic substances is responsible for this condition remains to be answered when the moot subject of rôle of uric acid in the production of disease is cleared up.

The disease which has received the special appellation of rheumatism of the skin is a dermatalgia without structural lesion, but the cutaneous disturbances, described above, are so frequently seen in those who give a clear history of rheumatic tendency that there can be very little doubt of the existence of a common cause.

Gout, which is a congener of rheumatism, is the cause ascribed to pruritic erythemata identical with those described in connection with rheumatism, but in addition there is a type of eczema chronic infiltrated with a tendency to warty excrescence, which is termed gouty eczema. Many have not considered it sufficiently characteristic to place it in a separate group. In both gout and rheumatism not only the cutaneous but the mucous surfaces are sometimes involved with the production of throat symptoms and bronchial asthma. This is accepted as evidence that both are susceptible in their own way to the action of a common irritant.

Gonorrhœa has been known to give rise to an irregular eruption of the type of congestive erythema, scarlatinaform in character and generally accompanying invasion of the serous surfaces of joints in what is known as gonorrheal rheumatism. This is capable of occurring independently of the administration of the balsams.

Diphtheria is not infrequently complicated with diffuse redness of the skin which resembles scarlatina and which is doubtless often confused with it, especially in the anginose types of the latter exanthem. It is of brief duration however, and is probably due to the reaction of the delicate skin of a child to the febrile movement. Antitoxine, which has come into vogue as routine treatment for diphtheria, is capable of producing eruptions upon the skin of an erythematous or urticarial or morbilliform type. The same is true of other serums, anti-tubercle, anti-streptococcus and the like. The eruptions are not especially characteristic and are vasomotor disturbances dependent somewhat upon personal idiosyncrasy. They may be general or appear about the seat of the infection, and sometimes the rash occurs as long as two weeks after the serum has been administered. According to Dubreuilh the serum of the horse when injected can produce a rash.

Renal disease is responsible for skin affections to a limited degree. Pruritus, urticaria, herpetiform dermatitis are sometimes associated with the small granular kidney or albuminuria, and certain patchy dermatitis or eczema with vesicles or bullæ, and followed by desquamation, may supervene as a uremic phenomenon according to Crocker and others. Deficient renal elimination is accepted as a cause of certain cases of eczema in the aged.

It must be borne in mind that the lowered vitality, associated with such a nutritional disturbance as accompanies disease and functional disorder of the kidney, renders the skin especially vulnerable to morbid influences.

Diabetes, another disturbance of the balance of nutrition associated with the kidney secretion, is held responsible for some dry pruriginous affections of the skin, and also for a condition characterized by patches of yellow, granular pigment which has received the name of xanthoma diabeticorum.

Hepatic and digestive disorders are capable of producing morbid changes in the skin. The two are so connected, that it is difficult to separate affections of the skin arising from liver complaints from those due to defective digestion and assimilation. The most frequent eruption in this connection is urticaria, which is due in most instances to the effect of intestinal toxins upon the vaso-motor and lymphatic systems. The influence of other toxic substances as leucomains and ptomains in the production of the disease is assumed rather than proven. Acne and eczema are other diseases considered to be maintained if not aroused by disorders of the digestive apparatus. The itching and staining of the skin are well known phenomena in the course of jaundice. These cutaneous affections are all assumed to be due to the retention of waste products which become autotoxic, under favorable conditions.

Malaria is not generally regarded as causative of skin disease beyond producing the appearance found in anemia and cachexia, and accostical herpes and urticaria; yet Engman, of St. Louis, and others have called attention to a purpuric and crusted eruption of the hands, which occurred in chronic paludism and which disappeared, or its disappearance was hastened, under antimalarial treatment. Two cases I have seen, which have answered to the description given by Engman, responded favorably to quinine and arsenic.

I shall not take up the frequent anomalies observed in Addison's disease, morphœa, leucoderma and the like, which belong to the class of trophoneurosis, in which the rôle of the toxins figure obscurely.

It is scarcely necessary for the present purpose to include a notice of the several external chemical, animal and mechanical irritants to which the skin is exposed, and which are capable of producing several types of eruption which subside when the irritant is removed. The best examples, because demonstrable a priori, of the reaction of the skin to toxic influences from within are the eruptions which sometimes follow upon the administration of certain drugs. Under these circumstances the eruptions are grouped under the general term of dermatitis medicamentosa. The occurrence and conduct of the eruptions are to a great degree governed by individual peculiarity, sex, age, climate, and such considerations as well as to the amount of the drug given and the length of time during which it has been administered. With some drugs one dose is sufficient to produce the eruption ; with others repeated doses, long continued, are required. The effect on the skin is brought about either by irritation produced by the drug in the process of elimination, or reflexly by its action on the nervous system—an angioneurosis.

The character of the eruption varies with the drug and length of administration. It may present all the primary efflorescences together with some of the secondary. It may be macular, papular, urticarial, vesicular, pustular, even necrotic when the cause is continuous.

The list of drugs having this action is a lengthy one, and I shall mention only a few of the most important. As has been pointed out by Morrow, those drugs which have the most effect upon the nervous system are those most prone to produce an eruption. It is well to remember that with the exception of two drugs, the bromine and iodine salts, there is nothing characteristic about the eruptions produced by drugs, and this must be carefully considered in making a diagnosis. Thus the eruption of belladonna resembles scarlet fever, while that of copaiba and cubebs is measly. The presence of the drug in the secretions and excretions as well as the abrupt appearance of rash when a drug capable of producing eruption is being taken and its vanishment on discontinuance of the drug are suggestive points in diagnosis.

The drugs which are in frequent use and which are capable of producing toxic effects as manifested by cutaneous rash are bromine and its compounds, quinine, antipyrine, belladonna, arsenic, the balsamic preparations, opium and its derivatives, mercury, the salicylates. Besides these there are many not in such frequent therapeutic requisition which can arouse a dermatitis medicamenta. The eruptions may simulate many diseases of the skin, but as a rule they fall in the class of the erythemata and are most likely due to an angioneurotic disturbance central or peripheral. The exceptions mentioned, bromine and iodine and their compounds are prone to produce acneiform or tubercular eruptions. Owing to the frequent employment of the salts of these metals for chronic affections the eruption is common and is observed in about seventy-five per cent. of all cases thus treated. The tendency to eruption is said to be diminished by changing to another salt of the metal or by administering arsenic simultaneously. After all it seems a matter of personal susceptibility, for those who have coarse, greasy skins develop the eruption quicker and with greater severity than those possessed of a skin of finer texture.

In the former class, if the drug be pushed beyond physiologic tolerance and continued exceedingly severe lesions of a fungating type may be produced. I have seen them in bromine and iodine when they resembled mycosis fungoides. Furuncular forms are occasionally seen, the furuncles prefering the hairy areas of the body. Destructive ulceration may take place. These changes are more apt to occur in connection with the bromine salts than with the iodine.

Iodism is manifested early in some individuals in an increase in nasal and salivary secretions, suffusion of the conjunctivæ, and often there is a certain amount of erythema apparent, with swelling of the face, especially the end of the nose, giving it a bulbous appearance. This is apt to be followed by a papulo-pustular eruption of greater or less severity and extent. From these more complex forms may proceed up to a high degree of severity—bullous, carbuncular, condylomatous or molluscoid. The papular and pustular phases of iodism may simulate smallpox.

Nodules will sometime appear. I recall a case of great disfigurement caused by them on the face of a woman who had been taking some "blood medicine" given her by an Indian doctor.

In comparing bromic and iodic eruptions, it will be observed that the eruption tends to centre around the sebaceous gland ducts and the hair follicles.

The recent occurrence in England of numerous cases of arsenical poison, due to its presence in beer, has given an excellent opportunity for observing the clinical manifestations.

Arsenical eruptions are not common except in such instances, where it is a chance adulteration of a beverage. Given internally, it sometimes produces a dermatitis papular, pustular, urticarial or even petechial. Boils or carbuncles occasionally occur. A general scarlatiniform rash, with irritation of the mucous surfaces, has been observed at times. A peculiar pigmentation, followed by desquamation, has been noted. The formation of callosities and clavus-like hypertrophies have been attributed to arsenic, which may become epitheliomatous.

Herpes is associated with the toxic effect of arsenic and is probably connected with its power of producing peripheral neuritis.

With argyriasm, plumbism and like pigment changes concerned with metallic deposits in the skin, I shall not engage your attention.

As was remarked at the outset, the question of toxic dermatoses is too unsettled and the subject too extensive to be more than approached in the present instance. The far-reaching significance of these dermatoses may perhaps serve to illustrate the importance of a proper study of dermatology and aid in placing it upon a high plane among the specialized fields of medicine.

